# 

**Published:** 1909-07-03

**Authors:** 


### BOOKS RECEIVED.

A. C. Fifield.

"Wastage of Child Life." By J. Johnson, M.D.

J. and A. Churchill.

"Southall's Materia Medica." By John Barclay, B.Sc., F.C.S.

The Caxton Publishing Company.

"The Edinburgh Stereoscopic Atlas of Obstetrics." Edited by G. F. Barbour Simpson, M.D., F.R.C.P.E., etc.

### THE GRADUATES' MEDICAL SCHOOLS.

DIARY FOR THE WEEK, JULY 5 to 10.

**ROYAL SOCIETY OF MEDICINE, 20 Hanover Square, W.**

**At 7.45 p.m.**

**July 8, Obstetrical and Gynecological Section.**

**At 3 p.m.**

**July 10, Therapeutical and Pharmacological Section,** at Cambridge.

**MEDICAL GRADUATES' COLLEGE AND POLYCLINIC, 22 Chenies Street, W.C.**

**At 4 p.m.**

**July 5,** Dr. Colcott Fox, **Skin.**
**July 6,** Dr. Harry Campbell, **Medical.**
**July 7,** Mr. Thomson Walker, **Surgical.**
**July 8,** Sir Jonathan Hutchinson, **Surgical.**
**July 9,** Mr. R. E. Bickerton, **Eye.**

**At 5.15 p.m.**

**July 5,** Dr. Purves Stewart, **Paralysis Agitans.**
**July 6,** Mr. Cecil H. Leaf, **Surgical Treatment of Cancer of the Rectum.**
**July 7,** Dr. A. F. Tredgold, **Feeble-minded Children.**
**July 8,** Dr. James Collier, **Acute Polyneuritis and Landry's Paralysis** (illustrated by cases).

**CENTRAL LONDON THROAT AND EAR HOSPITAL, Gray's Inn Road, W.C.**

**At 3.45 p.m.**

**July 6,** Dr. Atkinson, **The Nose.**
**July 9,** Dr. Dan McKenzie, **The External Ear.**

**LONDON SCHOOL OF CLINICAL MEDICINE, Seamen's Hospital, Greenwich, S.E.**

**At 2.15 p.m.**

**July 8,** Prof. Hewlett, **Artificial Immunisation in Infective Diseases.**

**At 2.30 p.m.**

**July 8,** Dr. Rankin, **Headaches and their Treatment.**

**NATIONAL HOSPITAL FOR THE PARALYSED AND EPILEPTIC, Queen Sq., Bloomsbury, W.C.**

**At 3.30 p.m.**

**July 6,** Dr. James Taylor, **Treatment of Nervous Diseases.**
**July 9,** Dr. Gordon Holmes, **Familial Ataxia—Friedreich's Disease.**

**THE HOSPITAL FOR SICK CHILDREN, Great Ormond Street, W.C.**

**At 4 p.m.**

**July 8,** Dr. Still, **Erythema Nodosum.**

**NORTH-EAST LONDON POST-GRADUATE COLLEGE, Prince of Wales's General Hospital, Tottenham, N.**

**At 4.30 p.m.**

**July 6,** Mr. J. H. Evans, **The Cesspools of the Body: Procedures of Surgical Drainage.**
**July 9,** Mr. R. P. Brooks, **Subconjunctival Injections in the Treatment of Eye Disease.**

**THE POST-GRADUATE COLLEGE, West London Hospital, Hammersmith, W.**

**At 10 a.m.**

**July 5 and July 8,** Surgical Registrar, **Demonstration.**
**July 9,** Medical Registrar, **Demonstration.**

**At 12 noon.**

**July 5,** Dr. Bernstein, **Pathological Demonstration.**

**At 12.15 p.m.**

**July 6,** Dr. Pritchard, **Practical Medicine.**
**July 7 and July 10,** Dr. Grainger Stewart, **Practical Medicine.**

**At 5 p.m.**

**July 5,** Mr. Bidwell, **Practical Surgery.**
**July 6,** Dr. Seymour Taylor, **Valvular Disease: Aortic Regurgitation.**
**July 6,** Dr. Pritchard, **Clinical Pathology.**
**July 7,** Dr. Beddard, **Medicine, V.**
**July 8,** Mr. Edwards, **Clinical Lecture.**
**July 9,** Mr. Armour, **Clinical Lecture.**

